# Hyperlipidemia: An Evidence-based Review of Current Guidelines

**DOI:** 10.7759/cureus.7326

**Published:** 2020-03-19

**Authors:** Karl T Clebak, Anthony B Dambro

**Affiliations:** 1 Family and Community and Medicine, Penn State College of Medicine, Penn State Health Milton S. Hershey Medical Center, Hershey, USA; 2 Family and Community Medicine, Penn State College of Medicine, Penn State Health Milton S. Hershey Medical Center, Hershey, USA

**Keywords:** cholesterol, treatment guidelines, statin safety, non-statin therapy, cardiovascular prevention, evidence-based

## Abstract

Cholesterol treatment guidelines have evolved in the United States from the 1988 Adult Treatment Panel (ATP) I, the ATP II guidelines, ATP III guidelines, the 2013 American College of Cardiology/American Heart Association guidelines, to the most recent 2016 recommendations from the United States Protective Services Task Force. The use of statins to treat hyperlipidemia has been widely accepted and recommended in adults aged 40-75 years old with at least one risk factor and a calculated 10-year cardiovascular disease risk of 10%. However, statin use is associated with myalgias, myopathy, musculoskeletal injury, liver injury, and increased diabetes risk. The evidence for non-statin treatments is mixed. Bile acid sequestrants and ezetimibe reduce cardiovascular events. There is no evidence that the addition of any fibric acid derivative to a statin improves cardiovascular outcomes. Available evidence suggests that the use of proprotein convertase subtilisin/kexin type 9 (PCSK9) enzyme inhibitors likely leads to little or no difference in mortality despite lowering lipid levels.

## Introduction and background

Summary of cholesterol treatment guidelines

The first United States cholesterol treatment guidelines, the Adult Treatment Panel I (ATP-I), were published in 1988 by the National Cholesterol Education Panel (NCEP). The focus was on primary prevention of coronary heart disease (CHD) by treating those with low-density lipoprotein (LDL) levels >190 mg/dL and no other risk factors to a goal of less than 160 mg/dL. If two risk factors were present or if CHD was already present, treatment should begin at >160 mg/dL and be reduced to 130 mg/dL or lower. [1] The second iteration of guidelines (ATP-II), published in 1993, furthered those recommendations and added a stricter goal for those with already established CHD of less than 100 mg/dL. ATP-II also introduced a triglyceride goal of <200mg/dL and added an HDL of less than 25 mg/dL as a new coronary risk factor. [1] A decade later, ATP-III further reduced the triglyceride goal (<150 mg/dL) and introduced risk stratification using a 9-step process. [2] The 2013 American College of Cardiology/American Heart Association guidelines base treatment decisions on risk, recommending statins for patients with known heart disease, an LDL ≥190 mg/dL, diabetics, and those with a 7.5% or higher 10-year risk of CV events. [3] In 2016, the United States Preventive Services Task Force recommended the use of statins in adults aged 40-75 years old with at least 1 risk factor and a calculated 10-year cardiovascular disease risk of 10%. [4] A comparison of current international guidelines for the treatment of cholesterol is summarized in Table 1 [1-5].

**Table 1:** Summary of international cholesterol guidelines LDL, low-density lipoprotein; LDL-C, low-density lipoprotein cholesterol; CKD, chronic kidney disease; CVD, cardiovascular disease References: [1-5]

	ATP-III	2013 American College of Cardiology/American Heart Association Guideline^3^	2011 European Society of Cardiology/ European Atherosclerosis Society Guidelines	2014 National Institute for Health and Care Excellence Guidelines	2012 Canadian Cardiovascular Society Guidelines
Risk assessment tool	Framingham Risk Score for Total CVD	Pooled cohort equations	SCORE risk assessment tool	QRISK2 risk assessment tool	Framingham risk score for total CVD
Specific LDL-C treatment targets	Yes	No	Yes	No	Yes
Lipid-lowering therapy for primary prevention	Yes LDL >190 mg/dL	LDL >190 mg/dL or LDL 70-189 mg/dL and 10-year risk > 7.5% 10-year risk < 7.5% and other factors	LDL >190 mg/dL or LDL <190 mg/dL and: 10 year risk > 10% moderate-severe chronic kidney disease and LDL>100 mg/dL LDL >115 mg/dL and risk factors	10-year risk >10 % or CKD	LDL >190 mg/dL or LDL <190 mg/dL and: 10 year risk > 20% 10-year risk 10%-19% LDL >75mg/dL 10 year risk 5-9% and LDL> 130 (optional) CKD or proteinuria High risk hypertension
Lipid-lowering therapy for primary prevention for those with diabetes mellitus	No	LDL >70 mg/dL	Type 2 and LDL >100mg/dL high-risk type 2 and LDL>70 mg/dL type 1 and target organ damage	Type 2 and 10-year risk >10% type 1 and age >40, duration of disease >10 years, nephropathy or CVD risk factors	Age >40 age <40 duration of disease >15 years age >30 and microvascular complications
Chronic kidney disease considered a high-risk factor	No	No	Yes	Yes	Yes

## Review

Statin safety

Myalgias/Myopathy/Musculoskeletal Injuries

Muscle complaints are a common occurrence in the outpatient setting among patients on statin therapy. In the cholesterol treatment trialists (CTT) meta-analysis, the risk of myopathy was found to be 0.5 per 1000 patients over five years equating to a number needed to harm (NNH) of 2000 [6]. In the large randomized controlled Heart Protection Study, participants were asked specifically about new or unexplained muscle pain or weakness at every 4-6 month follow-up [7]. At each follow-up visit, 6%-7% of participants reported such symptoms but at no point were there any significant differences between those receiving simvastatin compared with those on placebo [7]. By the end of the study, 32.9% of those on simvastatin and 33.2% on placebo had reported muscle pain at least once [7]. The incidence of myopathy during statin treatment in the SEARCH trial was found to be low. In patients taking simvastatin 80 mg daily, the incidence of a creatine kinase greater than 10 times the upper limit of normal was found to be 0.9% (53 of 6031) and 0.03% (2 of 6033) while taking simvastatin 20 mg daily [8]. The routine monitoring of creatine kinase levels is not recommended [9].

 A retrospective cohort study found increased musculoskeletal disorders and injuries with statin use; the number needed to harm for musculoskeletal disorders was found to be 47; the number needed to harm for musculoskeletal injuries was found to be 37 [10]. A cross-sectional analysis from the National Health and Nutrition Examination Survey database found the frequency of muscle complaints reported in usual care settings appears to be higher than in clinical trials with a frequency of 9% to 20% in outpatient settings [11]. It was also found that the prevalence of muscle pain in statin users is 50% greater than in non-users [11]. Statin users also have 50% to 60% greater odds of having musculoskeletal pain in the lower back and lower extremities. In absolute terms, this increase in muscle pain is 100 times greater than that reported in clinical trials [11].

Liver Injury

There has been mounting evidence of the low level of incidence of statin-induced liver injury. Most recently, the Federal Drug Administration (FDA) changed the required product labeling for statins [12]. The FDA had conducted several post-marketing reviews of statins and hepatotoxicity between 2000 and 2009. Those reviews consistently noted that reporting of serious statin-associated liver injury was extremely low with a reporting rate of ≤2 per one million patient-years [12]. Based on this data, the FDA no longer recommends routine periodic monitoring of serum alanine aminotransferase (ALT).

Diabetes Risk

A meta-analysis including 13 statin trials with 91,140 participants found that statin therapy was associated with a 9% increased risk for incident diabetes. [13] Data analysis found that the risk of development of diabetes with statins was highest in trials with older participants, but neither baseline body-mass index nor change in LDL-cholesterol concentrations accounted for variation in the risk. The number needed to harm over 4 years of statin therapy was found to be 255 [13]. The authors concluded that statin therapy is associated with a slightly increased risk of developing diabetes; however, the risk is low and when compared with the benefit in the reduction in coronary events clinical practice in patients with moderate or high cardiovascular risk should not change [13]. This modest potential risk of diabetes and the substantial benefit in the reduction of cardiovascular events should be used in the discussion to inform patients and guide shared decision making in clinical practice.

Evidence for non-statin therapies

There is limited evidence suggesting that the use of a bile acid sequestrant alone or in combination with a statin may reduce the incidence of cardiovascular events [14]. Ezetimibe, when used in combination with simvastatin, was found to have an additional small reduction in cardiovascular events [15]. In a meta-analysis of 11 randomized controlled trials including 6616 patients, the use of niacin was found to have a beneficial effect for secondary prevention of cardiovascular events, but the study was limited as the included trials varied in size and quality [16].

There has been no evidence that the addition of any fibric acid derivative to a statin improves cardiovascular outcomes [17]. The lack of clear evidence that supplementing statin therapy with triglyceride-lowering or HDL-raising drugs caused the FDA to withdraw approval of combination products containing a statin and fenofibric acid or niacin and are no longer available [18]. There has not been clear evidence for fish oil on primary or secondary prevention of cardiovascular outcomes. The new drug class proprotein convertase subtilisin/kexin type 9 (PCSK9) enzyme inhibitors, may further reduce LDL. However, available evidence suggests that PCSK9 inhibitor use likely leads to little or no difference in mortality [19].

## Conclusions

Cholesterol treatment guidelines have evolved in the United States from the 1988 ATP I, the ATP II guidelines, ATP III guidelines, the 2013 American College of Cardiology/American Heart Association guidelines, to the most recent 2016 recommendations from the United States Protective Services Task Force. The use of statins to treat hyperlipidemia has been widely accepted and recommended in adults aged 40-75 years old with at least 1 risk factor and a calculated 10-year cardiovascular disease risk of 10%. However, statin use is associated with myalgias (Number Needed To Harm of 2000), myopathy (0.9%), musculoskeletal injury (Number Needed To Harm of 37), liver injury (≤2 per one million patient-years), and increased diabetes risk (Number Needed To Harm over 4 years of statin therapy found to be 255). The evidence for non-statin treatments is mixed. Bile acid sequestrants and ezetimibe reduce cardiovascular events. There is no evidence that the addition of any fibric acid derivative to a statin improves cardiovascular outcomes. Available evidence suggests that the use of PCSK9 enzyme inhibitors likely leads to little or no difference in mortality despite lowering lipid levels.
